# The economic effects of perceptions of the Russia-Ukraine war in Ecuador

**DOI:** 10.12688/f1000research.131992.1

**Published:** 2023-06-19

**Authors:** Silvia Mariela Méndez-Prado, Julio Andres Medina-Castillo

**Affiliations:** 1 Faculty of Social Sciences and Humanities, ESPOL Polytechnic University, Guayaquil, Guayas, 090902, Ecuador

**Keywords:** Russia-Ukraine war, university students, economic perceptions, Ecuador

## Abstract

**Background:** Using an online questionnaire capturing the immediate economic and social effects of the Russia-Ukraine war. The study assesses the topics of more profound concern for university students and the variation of economic attitudes related to their socio-demographic variables.

**Methods:** Three hundred eighty-five participants, between 18 and 22 years of age, 49% female, leads us to identify significant differences by sex and economic status related to the stock crash, inflation, corruption, and poverty perceptions. However, the effect size and sampling could be improved.

**Results:** ANOVA confirms that the below-average economic status group feels more worried about higher inflation, while females tend to be more concerned about inflation, corruption, and poverty because of the conflict. Ordered logistic regression reveals that participants who express higher levels of concern regarding the impact of increased energy prices and poverty tend to exhibit greater overall worry.

**Conclusions:** Even though convenience sampling imposes constraints to extrapolate the results broadly, the research constitutes a benchmark for similar studies among Latin American and Caribbean countries since economic expectations and economic knowledge from citizens, applied in their decisions, play an essential role in national development.

## Introduction

Since the escalation of the Russia-Ukraine conflict, the commodities market volatility risk has increased due to Ukraine and Russia’s position as leading suppliers of food items, minerals, and energy such as oil, wheat, potassium, nitrogen, phosphorus, and gas ( Fang & Shao, 2022). Even though armed conflicts have always carried economic implications for the warring countries ( Christodoulakis, 2016), the Russia-Ukraine war may lessen the global GDP and increase inflation rates. Along the same lines, the worsening of this conflict would cause a reduction in household consumption due to rising utility bills, supply chain problems, economic growth obstacles and a decrease in investment ( Mbah & Wasum, 2022). Also, the sanctions imposed on Russia may have caused spillover effects, particularly an increase in systemic risk for the USA and its European allies ( Qureshi *et al.*, 2022).

Among Latin-American and Caribbean countries (LAC), Brazil, Chile, Colombia, Mexico, and Peru have experienced an impact on inflation since mid-2021 due to a surge in food and energy prices. In addition, the Russia-Ukraine war aggravated global oil and food price increases, leading to inflation. Moreover, as the conflict continues, a recession will likely impact Europe, reducing LAC’s exports and hindering growth in commodity-exporting countries.

As a LAC reference, the Ecuadorian agricultural imports, such as fertilizers and exports, mainly bananas and plantains, tuna, fish, and flowers, have decreased in Russia and Ukraine in 2022, while the Ukraine-Ecuador imports have been more volatile.

Previous studies have suggested that the public tends to identify internal economic crises that a country may undergo ( Berry & McDaniel, 2022). However, this judgment largely correlates with economic knowledge, which varies across socio-demographic cohorts ( Vicente & López, 2017). In that sense, we consider it pertinent to explore citizens’ economic perceptions and opinions regarding an external conflict that, *prima facie*, has nothing to do with their homeland and analyze whether these preconceptions are reflected in their responses. Moreover, under the premise that appraising economic issues stems from applying economic concepts and reasoning ( Walstad, 1997), economic perceptions would serve well as an unorthodox measure of economic knowledge in a developing country. They exert a noteworthy influence on households’ debt and asset management decisions ( Jappelli, 2010). Additionally, they benefit citizenship and decision-making on public issues ( Levstik & Tyson, 2008). Therefore, we exploit the fact that the conflict has had an impact on the Ecuadorian economy.

To the best of the authors’ knowledge, literature from Ecuador measuring university students’ viewpoints and perceptions of a momentous economic conjuncture is nearly non-existent. Therefore, the authors established a research question to fill the gap: What economic areas do Ecuadorians worry about the most due to the confrontation? Based on the research question, the study objectives are to identify if the economic perceptions and opinions vary depending on personal characteristics and to gauge the perception effects of several economic factors on students’ level of concern.

The following section provides a literature review of demographic factors that influence economic attitudes and economic indicators that represent a significant threat at the onset of war. It also presents hypotheses that can be draw from it to then influence the methods employed. Finally, the following results, conclusions, and discussions let us review the contribution and contextualized analysis.

## Literature review

### Demographic variation in economic attitudes

It is not beyond the realms of possibility that there would be differences across socioeconomic cohorts’ responses regarding their preoccupation with various economic topics since economic attitudes are in some way buttressed by economic knowledge ( Blinder & Krueger, 2004). Correspondingly, there is substantial evidence that sheds light on the discrepancies that can be found in economic literacy by sex, level of education, and income level. For example, males with higher education and income levels tend to grasp economic topics better ( Walstad & Rebeck, 2002). However, objective knowledge is not all that plays a role in people’s assessment of the economy. Political and personal gloom taints economic knowledge, including reporting estimates of economic indicators that are very far from being accurate, and involving personal economic distress (one’s personal situation), both of which tend to be reflected in the nature of an individual’s economic expectations ( Linsi *et al.*, 2022). Thus, we believe these personal circumstances are associated with the economic class students find themselves in.

In addition, political trust tends to be correlated with a prospective view of the economy (citizens judge the government by past economic shocks and, even more, their expectations). It is higher when the government can offer guarantees of economic growth ( MacKuen *et al*., 1992). Therefore, good levels of trust in the government could attenuate the uncertainties of war. For example, after the 2008-2009 global economic crisis, people from lower social status and with less education were strongly adamant about the competence of political institutions, which was conjectured as some resentment due to their post-crisis economic situation ( Dotti Sani & Magistro, 2016; Farvaque *et al*., 2017).

There is evidence of the relationship between trust and education level regarding the government, as more educated people initially favored the government. Still, that statement no longer holds true ( Dalton, 2005). Finally, LAC countries behave similarly. Citizens’ level of trust is positively correlated with income level, educational attainment, and the government’s economic and political performance.

### Economic aspects in war times

*Stock market*

Wars exert an apparent effect on the asset market. Major war events throughout history have resulted in structural breaks in both price movement and stock return volatility ( Choudhry, 2010). However, it is important to underline that changes in the stock market due to conflicts are heterogeneous and depend on how impactful the conflictive event is and the accuracy of people’s predictions regarding consequences ( Schneider & Troeger, 2006). In the same vein, the civil uprisings in the Arab World (Arab Spring) resulted only in increases in the volatility of Islamic indices. In contrast, international markets had no significant change ( Chau *et al*., 2014).

On the other hand, the onset of conflict may have generated more positive than adverse reactions in the stock market, in the sense that actual returns were higher than expected returns most of the time, possibly due to a war rally effect ( Guidolin & la Ferrara, 2010). In the context of the Russia-Ukraine conflict, the stock market reacted negatively before and after the launch of the Russian attack ( Boungou & Yatié, 2022). Countries like Russia, Poland, Hungary, and Slovakia responded in pre-event days, while the rest of Europe, India, Japan, and South Africa reacted in post-invasion days ( Ahmed *et al*., 2022; Yousaf *et al*., 2022).

*Supply-chain disruptions*

Globalization has advanced international fragmentation, which refers to allocating production blocks in different geographical areas to the public interest ( Jones & Kierzkowski, 2005). Evidence suggests that international fragmentation occurs not only with countries within the same region but has indeed escalated to global instances ( Los *et al*., 2015). Therefore, the effects of supply-chain disruptions have been felt internationally, despite being country-specific ( Arto *et al*., 2015; Carvalho *et al*., 2020). At the same time, supply chain disruptions are also associated with maritime transportation systems, as they represent over 90% of global trade. Therefore, the economic impact would be detrimental if the ports were coercively closed for any reason ( Pant *et al*., 2011; Thekdi & Santos, 2016). Thus, it is safe to posit that a significant conflict can have economic implications worldwide due to this supply-chain disruption threat and the interdependence of different countries in the production process for various goods that amount to a wealthy industry.

The Russia-Ukraine war has endangered the global supply-chain in different fields of the economy, including the automobile industry. Regarding the worldwide food supply-chain, researchers even point out that the conflict may reverberate in six critical aspects: production, processing and storage, transport logistics, food market retail, consumers, food-dependent services, and food quality ( Jagtap *et al*., 2022). In addition, COVID-19 has already impacted the supply-chain of recently recovered countries ( Aday & Aday, 2020). Consequently, it would not be unreasonable to suppose that the impact may be especially harmful.

*Energy prices*

Wars are periods that create energy supply uncertainty in consumers, depending on the region in which the conflict may occur. This uncertainty is then reflected in the ensuing prices during and in the aftermath of war ( Elder & Serletis, 2010; Lee *et al.*, 2021; Zhang *et al.*, 2022). At the same time, some authors have made a case for the triviality of the supply flow of crude oil, influencing its current price ( Kilian & Lutz, 2010; Wang & Sun, 2017). Others have remained neutral and have proposed the analysis period as the conciliator of the discrepancies in the oil supply’s significance in its price, where geopolitical events’ relevance start to wear off after 2000 ( Noguera-Santaella, 2016).

During the Russia-Ukraine conflict, the most affected region was Europe due to its dependency on Russian energy and the sanctions imposed on the country ( Mbah & Wasum, 2022). Still, it is not imprecise to say that the rest of the world is experiencing almost the same conditions and that they are struggling due to the increases in oil prices ( Appiah-Otoo, 2022; Surya Wicaksana *et al.*, 2022). In the case of LAC, countries in the region have been very dependent on liquid fuel imports for several years due to their necessity to supply the increasing consumption of a growing population and because of economic development ( Sheinbaum-Pardo & Ruiz, 2012). Consequently, it is to be expected that an increase in oil prices will directly affect liquid fuel prices depending on the fuel subsidy policy, which will vary across countries.

*Inflation*

During wars, governments must consistently increase their military expenses ( Bilmes, 2013). Still, when resources become scarce, they can only rely on international or domestic lending, which implies printing more money or abusing foreign exchange reserves ( Fischer & Easterly, 1990). Moreover, the literature corroborates this, whether the conflict affecting a country’s economy is internal or external. Regarding the Russia-Ukraine conflict, these two countries’ impact on the international energy and food supply is undeniable, which is demonstrated in the increasing global inflation ( Fang & Shao, 2022) that has previously been mentioned.

In addition to factual evidence of the incidence of wars on inflation, we need to consider how a layperson defines inflation in practical terms, since the public tends to overestimate it ( Georganas *et al*., 2014). In that sense, there seems to be inflation misconceptions that fall outside of what economics textbooks may state ( Leiser & Drori, 2005). Although these misconceptions could confound their concerns about higher inflation due to the war, students may still be preoccupied since inflation expectations are rooted in new information ( Armantier *et al*., 2013). In that regard, people are continuously bombarded with news and information through social media ( Gomez Rodriguez *et al*., 2014).

*Corruption*

The phenomenon of corruption tends to appear in the post-war period during the transition to peace ( le Billon, 2008; Lindberg & Orjuela, 2011). This harms countries’ economies by creating impediments to using their natural resources at their maximum capacity ( le Billon, 2014). At the same time, the post-war era can result in military dominance in the most extreme way, such as kleptocracy, which dilutes public funding due to its misuse by the government ( Waal, 2014). Although the connection between the shift from war to peace and corruption has been documented, there are cases where corruption was palpable during a conflict ( Wang, 2017), especially in the form of bribery between citizens and government representatives ( Ussr & Heinzen, 2007).

However, since corruption is described as actions from public officials aimed at their benefit and to the detriment of their institution’s purpose ( Rose-Ackerman, 2018), it seems plausible that politicians may take advantage of the crisis and act untruthfully for their benefit ( Caballero & Yared, 2010). In the same vein, political instability in the form of an external conflict can be considered a driver to more corruption ( Goel & Saunoris, 2017).

*Poverty*

During violent conflicts, it is only natural to expect the destruction of physical capital, as well as inefficiencies in matters of household productivity ( González & Lopez, 2007). In addition, evidence suggests how difficult it is to recover properly, especially when a household has been destroyed, or a portion of land has been lost ( Justino & Verwimp, 2013). Thus, the threat of conflict has a noticeable impact on household decisions and the coping mechanisms to which people must resort to ( Verpoorten, 2009).

Nevertheless, economic effects utterly depend on the violence experienced ( Serneels & Verpoorten, 2015). Authors even suggest that there could be benefits for the private sector in the form of protecting the national industry against the global market ( Mcdougal *et al.*, 2009). In the case of the Russia-Ukraine war, commodity price rises have impacted the poorest groups in the economy, and we suspect that respondents could be very uneasy about a surge in poverty.

### Hypotheses

Given the nature of the results, the following hypotheses were established:

H1. Differences in opinions would be found in economic status groups, sex, and educational attainment levels.

H2. Assuming that respondents guide their responses based on logic and available information, supply chain disruptions, higher energy prices, stock market crash, inflation, corruption, and poverty are responsible for a great level of student concern.

## Methods

### Sample and recruiting

The Ecuadorian data for the global study was collected by us, coordinated by the COVID-19 social science lab from the Faculty of Public Administration, University of Ljubljana (Grant No. P5-0093). Every country has a coordination research group and the national data collected belongs to the partner country. The survey addresses the economic and social impact that the Russia-Ukraine conflict may inflict on university students and it can be found in the extended data.

The recruiting was conducted using convenience sampling, contacting online representatives of universities in Ecuador, including representatives from ESPOL Polytechnic University. However, the sample also includes thirty-three students from other universities, such as the Pontifical Catholic University of Ecuador and Amazonian State University.

**Figure 1.**
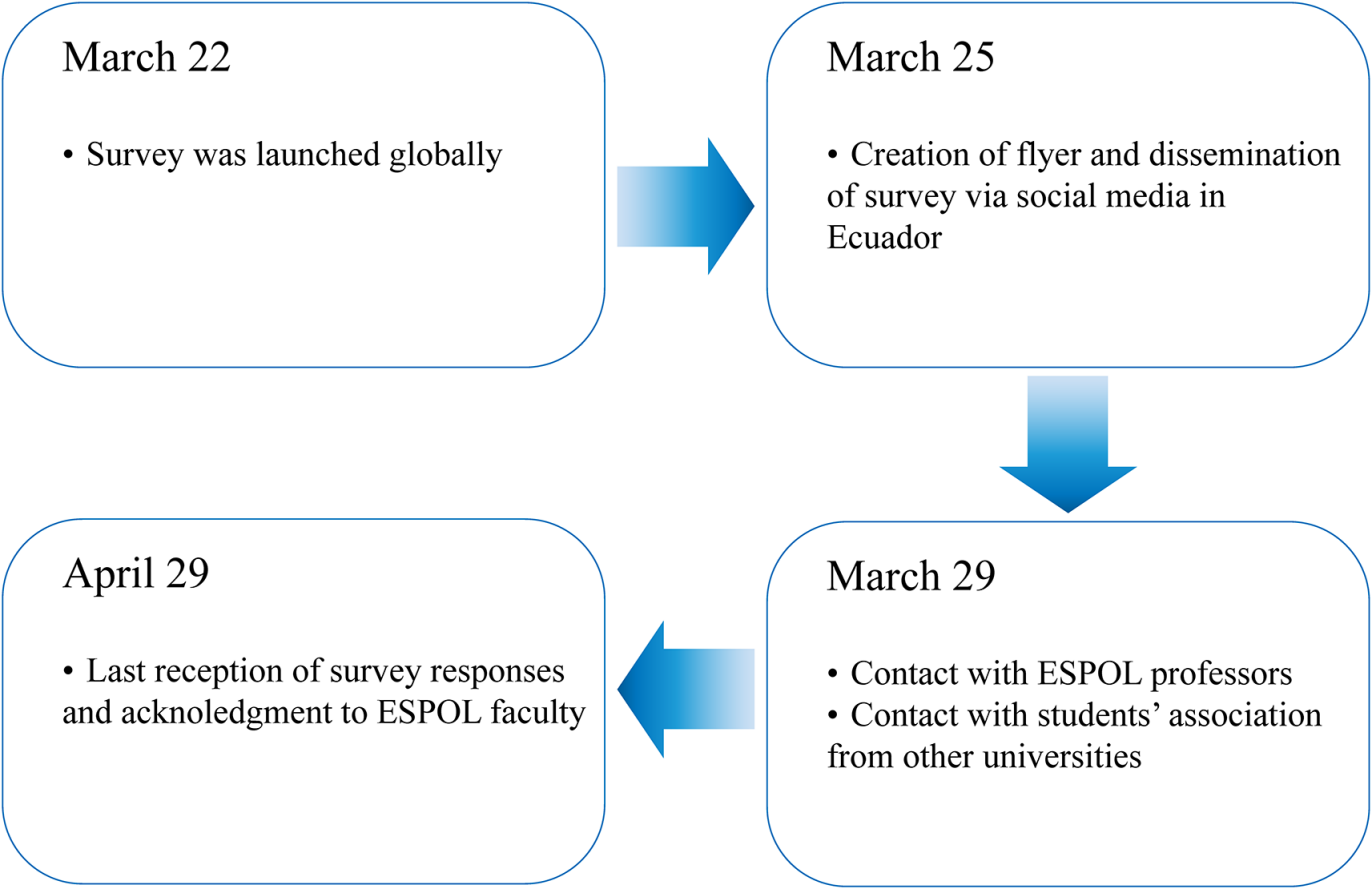
Dissemination process of the survey.

Figure 1 presents a summary of the main dates in the dissemination process. Although the survey was launched globally on March 22, the recruiting process in Ecuador began formally on March 25. The first thing to do was create a flyer containing information regarding the time it would take to complete it, the study’s objectives, the informed consent, and the anonymity of the responses. The flyer was mainly used as an instrument in social media to help respondents understand, with a glance at the flyer, the kind of questions they would be expected to answer and some context for the study.

The dissemination process was based on two main strategies, communication with students’ associations of different universities and with professors from ESPOL Polytechnic University during the extraordinary academic term on vacation time. From previous studies, we had access to a database with the contact information of student representatives from different universities in Ecuador. However, the process was unfruitful since students’ association elections are held every one or two years. As a result, the current students’ representatives were not acquainted with us, and further communication could not be established.

The second strategy was the one that produced most of the results captured in the survey. With the help of the ESPOL’s Registrar’s Office, we had access to a database that contained the names of 39 professors, the courses they were teaching, and the students enrolled in those courses. To obtain the most considerable response rate possible, we urged professors to make their students take the survey as an in-class activity. Since it was an online questionnaire, the phone’s browser offered the option to translate the questions via Google Translate to help students better understand the questions.

Twenty-one professors confirmed that their courses completed the questionnaire. Unfortunately, the rest did not since this academic term has a tight schedule, and professors must complete several activities and cover the material in their courses.

We can be confident that most responses came from this source, and the highest concentration of responses occurred between March 29 and April’s second week. The questionnaire was closed on April 29, obtaining 593 valid responses.

### Missing data

From the original 593 valid responses, there were several missing values. Therefore, after accounting for these missing data via listwise deletion for the variables considered in the study, the remaining responses were 410 (69% data remaining). Further steps to edit the database were: the PhD students’ responses only accounted for 0.84% of the original data (5 in total), which caused problems when we tested for the parallel line assumption that accompanies the ordered logistic model since there were no responses in each category of the general level of concern (some categories had 0 as frequency). Consequently, we opted to place PhD students in the category of postgraduates and continue with the analysis. A similar problem was encountered with Arts and Humanities students, their responses were around some specific categories, but not all categories were selected by them, leaving some of the responses with 0 as frequency. However, due to the mutual exclusivity nature of the study areas specified in the questionnaire, merging groups was not an option. Thus, we omitted their incomplete contributions resulting in 385 responses as the final dataset.

### Instrument

The COVID-19 social science lab conducted the survey in 2022 (for more information, see: http://www.covidsoclab.org/). It was initially designed in English, and students were informed of their voluntary and anonymous participation in the study. In addition, they certified being 18 or older and enrolled in a Higher Education Institution. Although the survey mainly focuses on students’ general perceptions regarding the military conflict, it included eleven sections corresponding to various topics detailed in Table 1. Demographic questions are presented in the form of multiple-choice, general reflections make use of open-ended questions, and the rest of the questions in the questionnaire were formulated as 5-scale Likert items. However, some presented an “I do not know” option. The original web-based survey was launched via the open-source web application 1KA.

**Table 1.** Sections of the questionnaire with number of questions.

Sections	Questions
S1 - Socio-demographic characteristics	1-7
S2 - General perception	8-10
S3 - Economic circumstances	11-14
S4 - Personal circumstances	15
S5 - Military and other circumstances	16-17
S6 - Actions and Sanctions	18-20
S7 - Satisfaction with response	21
S8 - Felt emotions	22
S9 - Post-war era	23-26
S10 - Additional socio-demographic characteristics	27-34
S11 - General reflections	35-36

As previously stated, we decided to analyze the economic perception of students of the Russia-Ukraine conflict. Thus, we only focused on section 3, “Economic circumstances”, but we also wanted to compare responses between socioeconomic levels. Therefore, we select sections 1, “Socio-demographic characteristics” and 10, “Additional socio-demographic characteristics”.

From section 1, we selected questions 3, 6, and 7, and from section 10, we chose questions 27, 28, and 32 regarding socioeconomic traits from the sample specified in Table 2.

**Table 2.** Descriptive Statistics.

	Mean	SD
Age (years)	21.27	3.38

	N	%
**Sex**		
Male	197	51.17
Female	188	48.83
**Level of study**		
Undergraduate	365	94.81
Postgraduate	20	5.19
**Field of study**		
Social Sciences	116	30.13
Applied Sciences	192	59.87
Natural and Life Sciences	77	20.00
**Community type**		
Urban	300	77.92
Suburban	51	13.25
Rural	34	8.83
**Economic status**		
Above average	23	5.97
Average	275	71.43
Below average	87	22.60
**Job status**		
Full-time	19	4.94
Part-time	103	26.75
No	263	68.31

From section 3, we selected questions 11 and 12. Within question 11, there were four items related to the services or goods price increases due to the war. Question 12 presented 11 items related to the level of concern regarding different economic indicators such as inflation, taxes, supply chain disruption, poverty, corruption, and stock crash, as seen in the survey in the extended data.

Socio-demographic questions from sections 1 and 10 are multiple-choice, while economic questions from section 3 are presented as Likert items.

### Data analysis

Data preparation, aggregation, and cleaning processes were performed using Stata/BE 17 software for Mac, as well as other statistical procedures that will be described shortly ( Liu, 2015).

*Kruskal-Wallis test and Mann-Whitney U test*

To test for differences in responses across groups, we used the nonparametric Kruskal-Wallis by ranks since we adopted the conservative attitude that Likert-items data are ordinal and not interval, i.e., options have a hierarchical order but consecutive points are not equally spaced, and also there is no need to assume normality distribution of answers but just homogeneity in variance, which was adequately met. Due to the nature of the Likert items, data has ties; therefore, we analyze the chi-squared adjusted for ties. On the other hand, when cohorts were distributed in two categories, we performed the Mann-Whitney U test, which is analogous to the Kruskal-Wallis. Tables were created using the program “asdoc” from Stata.

*Ordered logistic regression*

This model was chosen since it is common practice to model Likert-scale ordinal-type categorical dependent variables with ordered logistic regression ( Long & Freese, 2001). When $y^{*}$ represent the latent variable ranging from −∞ to + ∞ , then the structural model is:

$$y_{i}^{*}=x_{i}\beta +\epsilon_{i}$$

where β is a kx1 vector containing the parameters to be estimated, $x_{i}$ is a vector of exploratory variables containing characteristics of the population and $\epsilon_{i}$ is the random error. Then, the measurement model divides $y^{*}$ in J categories, which correspond to J−1 thresholds or cut points:

$$y_{i}=j\;\text{if}\;\tau_{j-1}<y_{i}^{*}<\tau_{j}\;\text{for}\;j=1,2,\ldots ,J$$

We also assumed that $\tau_{0}=-\infty$ and $\tau_{J}=\infty$ . The observed response categories were linked to a latent variable (general concern level) by the measurement model:

$$y_{i}=\left\{\begin{array}{l} 1↔\text{Not}\;\mathrm{at}\;\mathrm{all}\;\text{if}\;\tau_{0}=-\infty \leq y_{i}^{*}\leq \tau_{1}\\ 2↔\text{Slightly}\;\text{if}\;\tau_{1}=-\infty \leq y_{i}^{*}\leq \tau_{2}\;\\ 3↔\text{Somewhat}\;\text{if}\;\tau_{2}=-\infty \leq y_{i}^{*}\leq \tau_{3}\\ 4↔\text{Moderately}\;\text{if}\;\tau_{3}=-\infty \leq y_{i}^{*}\leq \tau_{4}\;\\ 5↔\text{Extremely}\;\text{if}\;\tau_{4}=-\infty \leq y_{i}^{*}\leq \tau_{5}=+\infty \end{array}\right.$$

Since the model relies on the parallel line’s assumption, we tested for it using the Brant test ( Brant, 1990). The null hypothesis tests the hypothesis that the ordered logistic regression is valid. We used “spost13” from Stata and found no evidence to reject the null hypothesis at no conventional significance percentage. We, therefore, continued with its analysis. In addition, the elaboration of tables was made possible through the program “outreg2” from Stata ( Liu, 2015).

### Regression analysis consideration

The ordered logistic regression provides some guidance with the interpretation of parameters, but it is not interpreted as causality since it requires other assumptions not discussed here.

### Ethical approval and consent

This study followed the research protocol of the Declaration of Helsinki. We requested that the Dean of Research of ESPOL Polytechnic University created the Ethics committee and we obtained certification concerning their approval to proceed with the data online collection. All participants signed informed consent forms at the beginning of each online questionnaire (see the informed consent in the survey in the extended data). The questionnaire provided the University of Ljubljana researchers’ contact details for further questions that participants may have. This section from the questionnaire ended by indicating to participants that, by advancing to the next page, they certify being informed of the voluntary and anonymous nature of participation in this study, that they are 18 or older and that they are currently enrolled in a higher education institution.

## Results

### Participants’ description

First, we present some descriptive statistics in Table 2 regarding the study’s sample ( Méndez-Prado *et al.*, 2023). As it is depicted, most respondents were male (51.17%) with an average age of 21, pursuing a bachelor’s degree (94.81%) in the field of Applied Sciences (59.87%). In addition, it should be noted that over 75% of the respondents live in an urban community, and over 70% described themselves as average regarding economic status. Finally, as most students are undergraduates, they are not likely to have full-time jobs (5.18%). The data came from students’ self-report.

### Kruskal-Wallis test and Mann-Whitney U test

Table 3 presents a summary of the concern levels between the economic topics that were previously discussed and which cohorts presented significant differences. In addition, specific responses across groups are shown in the extended data ( Méndez-Prado *et al.*, 2023).

**Table 3.** Differences on preoccupation levels regarding economics topics by socio-demographic cohorts.

	Stock crash	Supply chain problem	Higher energy prices	Higher inflation	Higher corruption	Higher poverty
**Sex**						
Male	2.286 **	1.217	0.819	2.053 **	2.068 **	3.113 ***
Female						
**Level of study**						
Undergraduate	1.076	1.901 *	0.715	0.616	-1.032	0.428
Postgraduate						
**Field of study**						
Social Sciences	1.141	1.18	0.78	0.801	0.158	0.321
Applied Sciences						
Natural and Life Sciences						
**Community type**						
Urban	2.103	0.036	1.237	1.62	3.53	1.221
Suburban						
Rural						
**Economic status**						
Above average	5.61 *	2.328	2.402	7.875 **	4.546	6.488 **
Average						
Below average						
**Job status**						
Full-time	0.568	1.03	0.813	0.306	1.339	0.422
Part-time						
No						

Note: Z-score for groups of two categories (Mann-Whitney U test) and H-statistic for groups of 3 (Kruskal-Wallis test).

*** p < 0.01.

** p < 0.05.

* p < 0.1.

Stock crash, inflation, corruption, and poverty were statistically significant, while the other variables, such as supply chain problems and energy prices, were not; more details about the socio-demographic cohort and the corresponding variables are presented below.

*Stock market*

Responses regarding a possible stock crash due to the Russia-Ukraine conflict seem to be in respondents’ minds in the different groups formed. There are almost no differences in the responses between groups. However, there are differences in the level of preoccupation between females and males $\left(Z\left(N=385\right)=2.286,p=0.022,\eta^{2}=0.014\right)$ , the probability of females being more worried than males about a stock market crash is 56.5%.

*Supply chain disruption*

As in the first question assessed, most responses were concentrated between the “Somewhat” and “Moderately” levels of concern towards a supply chain disruption. Regarding the direction the responses tend to follow, they were similar to the overall result. On this occasion, no significant statistical differences were found across groups.

*Energy prices*

Responses regarding this topic were also between “Somewhat” and “Moderately” but more inclined towards the latter of a potential stock market crash or supply chain disruption. As in the previous section, we did not find significant statistical differences across the socio-demographic levels. Nevertheless, something to highlight is that, unlike earlier questions in which the second most frequent response was feeling “somewhat” concerned, students reported a sentiment of “extremely” concern as the second most common answer.

*Inflation*

The trend of responses regarding inflation also falls between “Somewhat” and “Moderately”. The Mann-Whitney test revealed differences in concern levels about inflation between males and females $\left(Z\left(N=385\right)=2.053,,\text{p}=0.04,,\eta^{2}=0.011\right)$ , the probability of females feeling more worried than males about inflation is 55.8%.

Furthermore, the Kruskal-Wallis test showed differences in concern levels about inflation among the economic group students they were part of $\left(\chi^{2}\left(2,N=385\right)=7.875,p=0.02,E_{R}^{2}=0.021\right)$ . A posthoc test using Dunn’s test with Bonferroni correction showed differences between the below-average group and average group $\left(p=0.017\right)$ , and between the below-average group and above-average group $\left(p=0.051\right)$ . In both cases, it seems that the below-average group is more concerned about inflation due to the war.

*Corruption*

The topic of corruption does raise more concerns in students in a more tangible way than in previous sections. Responses were more inclined towards the option “Extremely.” Furthermore, there were differences between males and females $\left(Z\left(N=385\right)=2.068,p=0.038,\eta^{2}=0.011\right)$ , the probability of females feeling more worried than males about corruption is 55.8%.

*Poverty*

Poverty is the topic that most students are more concerned about, and most responses favor the option “Extremely.” However, there are significant differences across cohorts. For instance, we found differences between males and females regarding their concern with poverty $\left(Z\left(N=385\right)=3.113,p=0.002,\eta^{2}=0.025\right)$ , the probability of females feeling more worried than males about poverty is 58.7%.

In addition, the Kruskal-Wallis test revealed that economic status had some effect on how much concern students regarded the topic of poverty $\left(\chi^{2}\left(2,N=385\right)=6.448,p=0.039,E_{R}^{2}=0.017\right)$ . A posthoc test using Dunn’s test with Bonferroni correction showed differences between the below-average group and average group $\left(p=0.046\right)$ , and between the below-average group and above-average group $\left(p=0.054\right)$ . The below-average group is more concerned with poverty than the other two.

### Ordered logistic regression

To examine which economic phenomena were associated with a more significant concern response, we performed an ordered logistic regression, and results for three different specifications are presented in Table 4.

**Table 4.** Ordered Logistic Regression for economic perceptions influencing general level of worry.

Variables	Model A	Model B	Model C	Odds ratio Model A	Odds ratio Model B	Odds ratio Model C
Worry						
Age	0.0429 (0.247)	0.0380 (0.281)		1.044 (0.247)	1.039 (0.281)	
**Sex**						
Male	-0.148 (0.5)	-0.115 (0.59)		0.863 (0.5)	0.891 (0.59)	
**Study level**						
Postgraduate	-0.268 (0.558)	-0.183 (0.68)		0.765 (0.558)	0.833 (0.68)	
**Study area**						
Social Sciences	-0.319 (0.282)	-0.297 (0.306)		0.727 (0.282)	0.743 (0.306)	
Applied Sciences	-0.306 (0.26)	-0.326 (0.226)	-0.204 (0.294)	0.737 (0.26)	0.722 (0.226)	0.815 (0.294)
**Community type**						
Urban	0.138 (0.708)	0.000986 (0.998)		1.148 (0.708)	1.001 (0.998)	
Suburban	0.457 (0.313)	0.318 (0.473)	0.389 (0.186)	1.580 (0.313)	1.375 (0.473)	1.475 (0.186)
**Economic status**						
Below average	0.0976 (0.701)	0.170 (0.49)		1.102 (0.701)	1.186 (0.49)	
Above average	0.196 (0.652)	0.104 (0.807)		1.217 (0.652)	1.109 (0.807)	
**Job**						
Full-time	-0.206 (0.702)	-0.153 (0.769)		0.813 (0.702)	0.858 (0.769)	
Part-time	-0.438 * (0.065)	-0.433 * (0.063)	-0.370 * (0.089)	0.646 * (0.065)	0.648 * (0.063)	0.691 * (0.089)
Energy prices	0.164 (0.218)	0.222 * (0.057)	0.283 *** (0.005)	1.179 (0.218)	1.248 * (0.057)	1.327 *** (0.005)
Essential goods prices	0.110 (0.439)			1.116 (0.439)		
Essential services prices	0.0335 (0.807)			1.034 (0.807)		
Luxury goods prices	0.0481 (0.703)			1.049 (0.703)		
Luxury services prices	-0.0144 (0.901)			0.986 (0.901)		
Stock market crash	0.0255 (0.861)	0.0934 (0.463)		1.026(0.861)	1.098 (0.463)	
Inflation	0.148 (0.391)	0.0569 (0.692)		1.159 (0.391)	1.059 (0.692)	
Taxes	-0.116 (0.498)			0.891 (0.498)		
Unemployment	-0.378 ** (0.011)			0.685 ** (0.011)		
Saturation EU labor force	0.128 (0.362)			1.137 (0.362)		
Limited international trade	0.290 * (0.086)			1.336 * (0.086)		
Supply chain problems	-0.0996 (0.527)	0.0791 (0.564)		0.905 (0.527)	1.082 (0.564)	
Poverty	0.297 * (0.086)	0.168 (0.302)	0.281 *** (0.008)	1.346 * (0.086)	1.183 (0.302)	1.324 *** (0.008)
Corruption	-0.0271 (0.853)	-0.0408 (0.769)		0.973 (0.853)	0.960 (0.769)	
Crypto market crash	0.122 (0.223)			1.130 (0.223)		
Cut1	-0.547 (0.960)	-1.066 (0.919)	-1.643 *** (0.513)	0.579 (0.555)	0.344 (0.316)	0.193 *** (0.0992)
Cut2	0.915 (0.927)	0.364 (0.883)	-0.211 (0.451)	2.496 (2.314)	1.440 (1.271)	0.810 (0.365)
Cut3	2.646 *** (0.929)	2.050 ** (0.884)	1.466 *** (0.454)	14.10 *** (13.10)	7.767 ** (6.866)	4.332 *** (1.967)
Cut4	5.258 *** (0.964)	4.581 *** (0.913)	3.968 *** (0.494)	192.1 *** (185.2)	97.57 *** (89.11)	52.88 *** (26.12)
Observations	385	385	385	385	385	385
Pseudo R-squared	0.0557	0.0375	0.0324			
Log Likelihood value	-463.4	-472.3	-474.8			
Degrees of freedom	26	17	5			
Chi-squared	54.69	36.83	31.78			

Note: The section of cuts represents the estimated threshold parameters of the latent variable. For instance, cut one is the cutoff point that separates the response “Not at all” from “Slightly” feeling worried. For the stepwise selection process, covariates are eliminated based on a predefined significance threshold level: the alpha-to-drop is set to 0.3. P-values in parentheses for coefficients and Standard errors for cutoffs.

*** p < 0.01.

** p < 0.05.

* p < 0.1.

The base categories for sex, study level, study area, community type, economic status, and job status are female, undergraduate, rural, average, and no job, respectively.

Model A includes all socio-demographic and economic questions from the questionnaire. Model B includes socio-demographic variables and only the economic topics that were discussed previously. Model C only shows variables selected via stepwise selection, resulting in a chi-squared ( $\chi_{\left(5\right)}^{2}=31.78^{***}$ ) that is statistically significant at 1%, although the pseudo $R^{2}$ is not exceptionally high in any specification.

At the same time, we estimated the odds ratio for both models to shed light on the interpretation of the results. In Model A, statistically significant coefficients correspond to the “Unemployment” category at 5% and both “limited international trade” and “poverty” at 10%. On the other hand, Model B presents “higher energy prices” as statistically significant but only at the 10% level. Its coefficient differs from the one estimated in Model A. Model C shows both “higher energy prices” and “poverty” as statistically significant at even the 1% level, although the coefficient varies across specifications. The coefficient for “higher energy prices” is more like the one in Model B, while the coefficient for “poverty” is almost the same as in Model A.

Coefficients can be interpreted as follows: a one-unit increase in the predictor changes the odds of feeling more worried by the coefficient in percentage points when holding all the other predictors constant. At the same time, the odds ratio reflects the magnitude by which economic topics cause students to worry more. Suppose the odds ratio is greater than 1. In that case, the topic associated with that odds ratio increases their concern level, and an odds ratio smaller than one refers to the opposite.

For example, going one level up in the concern level scale about poverty will increase the odds of feeling more worried by approximately 32% to 35% when holding all the other predictors constant. Moreover, a higher level of concern about higher energy prices is associated with an increase likelihood of feeling generally preoccupied, ranging from 25% to 32%, when holding all the other covariates constant. For this reason, the economic indicators students think are more distressing are “poverty” and “higher energy prices.” Nevertheless, a particularly odd coefficient was the one that accompanies unemployment. Therefore, if we follow the previously established structure, we will claim that going one level up in the concern scale about unemployment will decrease the odds of feeling more worried.

## Discussion

It is entirely reasonable that students feel preoccupation concerning the Russia-Ukraine conflict since there are noticeable symptoms in different sectors of the economy that not only affect countries in Europe but also Ecuador and the rest of the world ( Ahmed *et al.*, 2022; Jagtap *et al.*, 2022; Mbah & Wasum, 2022; Yousaf *et al.*, 2022).

Results showed that responses across different socioeconomic levels were homogeneous according to the general student response. We are inclined to believe this is due to the nature of the Likert items since the only possible options are within the constraint of 5 levels, significantly limiting the variability of answers across groups. However, there were specific cases where differences resonated between the groups divided by sex and economic status. Differences in levels of worry by sex are in line with the literature. For example, females tend to feel more insecure about economic evolution, which is consistent with the literature regarding inflation. In contrast, males are more well-acquainted with financial information ( Walstad & Rebeck, 2002) and feel more optimistic about the temporality of the war event.

Along the same lines, a more significant preoccupation with topics such as stock market crashes, corruption, and poverty could be explained by how females tend to be more distrustful of economic institutions ( Farvaque *et al.*, 2017).

On the other hand, differences found in economic groups could be explained by the magnitude in which inflation and poverty influence the below-average group’s decision-making and the constraints it would create for earning a living. Likewise, this type of reasoning is consistent with the tendency to reflect one’s current situation in economic opinions and attitudes ( Linsi *et al.*, 2022). Thus, it should be credible that members of the below-average group would worry more about inflation and poverty since they already have experienced difficulties coping with the current economy.

Although differences across economic cohorts came to be expected according to H1, we do not feel positive to support that claim since the number of people constituting the above-average and below-average categories were not particularly large and comparable to the large amount that identified themselves as average. Therefore, we cannot posit if the differences can be explained due to the relationship between knowledge and economic status or the lack of participants that could fill in the other categories. Furthermore, since the groups of females and males were more balanced, we feel more optimistic about the differences found across them.

Regarding the ordered logistic regression, most covariates were not considered statistically significant, not even at 10%, for Model A and Model B. Model C, did find more statistically significant coefficients at 1%, and their values did not differ substantially across specifications. Overall, coefficients in at least two models estimate that “higher energy prices” plays a vital role in students’ worry level, and the same occurs with the category of “poverty”. H2 was partially supported by evidence found in the ordered logit regression. Still, more than half of the proposed economic topics did not represent an effect on the preoccupation level of students that was not statistically different from zero. As we have discussed, the impact of war on poverty is very substantial, in addition to the global consequences that this conflict has on staple goods, which could only worsen the situation of many citizens.

On the other hand, energy prices like oil prices tend to be in the mind of most laypeople, and it is only natural that an increase in these prices results in their discomfort. Other topics, such as a stock market crash and supply chain disruption, could be considered more sophisticated. It is not likely that students in the sample would be very aware of the impacts Russia-Ukraine can exert in those aspects. We would have expected topics such as inflation and corruption to be along with students’ critical drivers of preoccupation. However, that does not seem to be the case which could indicate an endogeneity problem in our models that is confounding our estimates.

### Contributions

The literature examining economic perceptions from the LAC region toward an external economic event that has important implications across the globe is scarce. In that sense, this paper corroborates apparent differences in students’ economic attitudes regarding a significant conflict. Also, responses serve as an unorthodox measure of students’ economic knowledge if we consider how objective knowledge makes part of their judgment. Literature has reported that decision is not knowledge-based solely ( Linsi *et al.*, 2022), we contribute to that statement since it is clear that a vast majority of economic topics were ignored by respondents even though the war has a major impact on them, which is to say that there are other aspects like socio-demographic characteristics or other unmeasured variables that condition their opinion. In addition, we offer some insights about how well respondents detect the economic consequences of war for their own country when the corresponding conflict affects markets and prices globally due to other circumstances, even though the respondents’ country does not participate directly in the conflict.

Moreover, we gauge students’ level of concern about inflation due to the war, e.g., we provide, to some extent, inflation expectations which are vital in predicting the actual inflation rate. Furthermore, responses were found to reflect the news coverage and the dissemination of information that students had access to during the escalation of the war. Finally, results also advocate that the government should consider financial education strategies for their citizens since it is clear that not all cohorts have a good grasp of the functioning of the economy and that knowledge is a key driver of economic growth.

### Limitations and future work

The first limitation that needs to be addressed is the amount of missing data that initially composed the sample. A reduction of almost 35% of the sample reduces the statistical power the analysis could have offered regarding differences in responses across cohorts and the association of other economic indicators’ preoccupation with the general level of worry. In addition, a problem arising from the composition of Likert items is that options do not include a mid-point where respondents can express their neutral opinion or indifference, which could be appropriate given the nature of the latent variable we are trying to gauge. However, when faced with a coarse scale (a scale with fewer points), it is better not to include a mid-point since respondents can use it as a dumping ground or conceal the person’s actual response ( Chyung *et al.*, 2017). In addition, socioeconomic characteristics could have made use of a better scale. For example, economic status could have included more distinguishing categories, considering that “average” seems ambiguous at best. Besides the economic status question, other socioeconomic cohorts were not remarkably balanced, for example, by job status, the area in which students live, and their educational attainment, for which responses were concentrated in the option “No job”, “Urban area” and “undergraduate” respectively. This results in an analysis in which it is very cumbersome to distinguish differences between the cohorts. In addition, cases with a significant difference demonstrated low Epsilon-squared and Eta-squared coefficients, which represent the effect size of how cohorts could explain the variance on the dependent variables.

Moreover, a major drawback is also the sampling technique that was employed. Convenience sampling does not permit extrapolation of the current results to a general population, making them only applicable to this sample ( Stratton, 2021). Consequently, it is noteworthy that most respondents came from ESPOL Polytechnic University, which does not constitute a representative sample of Ecuadorian university students.

On the other hand, the ordered logit analysis had pseudo $R^{2}$ statistics that were very low across different specifications, which translates into the models’ incapacity to explain the relationship between the predictors and the level of worry. This could stem from the fact that there is endogeneity between the variables that were considered in the models.

Future research might overcome these limitations by obtaining a bigger and more representative sample outside ESPOL Polytechnic University since the amount of deleted data due to missing information was very high relative to the total sample, undermining some statistical procedures. At the same time, the sample obliviates those students who do not have internet access and are from lower social status groups. Similarly, demographic questions could have been better specified not to introduce bias to individual responses. Finally, due to convenience sampling, the recruiting process could also resort to snowball sampling to obtain more student responses ( Audemard, 2020). However, simple random or stratified sampling would be ideal for extrapolating conclusions.

### Other implications

Governments should be very aware of the economic knowledge their citizens have and propose financial education programs that fill the gaps of understanding of some economic topics, which in turn can be very beneficial for democracy since a more educated society can assess more effectively the economic decisions that are being taken and act more actively in democracy ( Davies, 2015). In addition, economic knowledge can urge the acceptance of not-so-popular policies if they are deemed necessary for prosperity ( Huston, 2012). Moreover, understanding how the public perceives the economy is relevant for interpreting how the economy will behave ( Roos, 2008; Roth & Wohlfart, 2019). Additionally, understanding citizens’ level of worry will allow the government to anticipate potential discontent later, which can be expressed through protests. In relevance to this topic, inflation expectations, which are measured in the form of level of concern, are nothing short of a relevant characteristic that needs to be studied for monetary policy purposes ( Armantier *et al.*, 2013). However, due to the previously reported limitations, we believe the questionnaire could be improved for further research on attitudes.

## Conclusion

The Russia-Ukraine war has led many to grow uneasy due to the economic and political implications that its development may have on the world. In this respect, this study provides some insight from Ecuadorian university students regarding their preoccupation with the conflict. We found no significant differences across some cohorts formed by study area, educational attainment, community type, or job status at any convention level of significance. However, there were some differences across economic groups and by sex, in which people from the below-average group tended to feel more worried about imminent higher inflation. At the same time, females also felt more concerned about inflation and corruption and that the poverty rate will increase because of the conflict. Furthermore, an ordered logistic regression was applied to measure the economic phenomena that cause the most disruption in the level of preoccupation of students. The results showed that students were more worried about higher energy prices and poverty because of the war tend to exhibit a more considerable sense of general preoccupation.

Results in the paper can lead the way in assessing students’ preoccupation regarding an external military conflict, which is of particular importance for policymakers that depend on the trust that citizens put in them. It is their job to offer reassurance in response. Additionally, we recognize the implication that expectations have in a country’s economy and serve as an unorthodox metric of citizens’ economic knowledge, which is particularly important for a better democracy and effective public policies.

However, there is still room for improvement regarding the recruiting process as it could have collected more responses to offset the missing data we found and improve the statistical results. Furthermore, results cannot be extrapolated in other contexts due to the nonprobability sampling technique. In addition, coefficients from the ordered logistic regression analysis tend to vary across specifications. However, the study points to a general sense of preoccupation respecting the war, at least for Ecuadorian citizens. This can constitute a starting point for other researchers to conduct investigations, considering all suggestions discussed previously, in Latin America.

## Data Availability

Mendeley Data: University students’ perceptions about the Russia-Ukraine war. A global survey with Ecuadorian dataset. https://doi.org/10.17632/ryghfbpnd4.2 ( Méndez-Prado *et al., *2023) This project contains the following underlying data:
- 280323 Datasets Ecuador - Russia-Ukraine war 2022.xlsx (Ecuadorian students’ responses to the questionnaire launched by the Faculty of Public Administration, University of Ljubljana from Slovenia [with international academic partners]). - 280323 Questions R-U WAR.pdf (The original survey that includes all sections). Mendeley Data: University students’ perceptions about the Russia-Ukraine war. A global survey with Ecuadorian dataset. https://doi.org/10.17632/ryghfbpnd4.2 ( Méndez-Prado *et al*., 2023) This project contains the following underlying data:
- Frequency tables of level of concern related to economic topics by socio-demographic group.pdf (Specific responses to the economic questions that were analyzed divided by socio-economic cohorts). - Students’ perception on the Russia-ukraine war 2022 survey.pdf (Original survey from the webpage with the informed consent and the options for each question). Data are available under the terms of the Creative Commons Attribution 4.0 International license (CC-BY 4.0).
