# Supplementary material for: The economic effects of perceptions of the Russia-Ukraine war in Ecuador
Source: F1000Res. 2023 Nov 21;12:701. Originally published 2023 Jun 19. [Version 2] doi: 10.12688/f1000research.131992.2 (PMC10565421; doi:10.12688/f1000research.131992.2)
Supplement: Peer review history

# Reviewer report: Reviewer response for version 2

**DOI:** 10.5256/f1000research.157560.r224423

**Published:** 2023-11-22

**Authors:** Luisa Langer

**Affiliations:** 1 Universität Mannheim Fakultät für Betriebs­wirtschafts­lehre, Mannheim, Germany

I have no further comments to make. The revised article is in good shape and the authors should be commended for their great efforts in improving the paper.

Is the work clearly and accurately presented and does it cite the current literature?

Yes

If applicable, is the statistical analysis and its interpretation appropriate?

Yes

Are all the source data underlying the results available to ensure full reproducibility?

Yes

Is the study design appropriate and is the work technically sound?

Yes

Are the conclusions drawn adequately supported by the results?

Yes

Are sufficient details of methods and analysis provided to allow replication by others?

Yes

Reviewer Expertise:

Governance

I confirm that I have read this submission and believe that I have an appropriate level of expertise to confirm that it is of an acceptable scientific standard.

# Reviewer report: Reviewer response for version 1

**DOI:** 10.5256/f1000research.144886.r204347

**Published:** 2023-10-10

**Authors:** Matti Kohonen

**Affiliations:** 1 Financial Transparency Coalition, London, UK

The study is well presented, and methodologically sound in terms of the sample size and sampling method as part of an international study replicated in multiple countries. The causal effects of the Russia-Ukraine war could be discussed further in the case of Ecuador, along with the feedback mechanisms, and various ways in which we see the impacts arising in Ecuador. For instance, while there are no businesses by sanctioned oligarchs in Ecuador (this should be discussed if there are), Russian and Ukrainian companies must have had some presence in the Ecuadorian economy. E.g. Russian fishing fleets, mining companies, or other financial interests. Also sanctions are being evaded actively, and again while Ecuador is not a location where sanctions evasion takes place (rather Middle-East, parts of Asian tax havens), or supply-chain diversions (Kazakhstan, and other Central Asian border states with Russia), there could be an upsurge in trade with Ecuador with say Kazakhstan and Georgia that is noticeable in trade statistics already. However, even adding these elements, the most likely impact is higher global inflation and increase in the price of fertilisers which LAC countries import from Russia / Belarus where fertilisers are produced for the world market. Grain prices have increased, which may help commodity exporters (some LAC Countries), but Ecuador exports tropical fruits rather than grains so this won't help Ecuador directly. Grain for cattle feed may have increased in price, making beef production more costly.

 In terms of the attitudes, I am no statistician to check the validity of the argument in drawing the statistical and regression analysis, and I have noted this. I can say that the attitudes are somewhat what I'd expect, it's worth adding that women may be have a lower income and thus due to their lower socioeconomic position they may feel more worried about poverty and inflation, so this is could be mentioned in terms of analysing the results.

Is the work clearly and accurately presented and does it cite the current literature?

Yes

If applicable, is the statistical analysis and its interpretation appropriate?

I cannot comment. A qualified statistician is required.

Are all the source data underlying the results available to ensure full reproducibility?

Yes

Is the study design appropriate and is the work technically sound?

Yes

Are the conclusions drawn adequately supported by the results?

Yes

Are sufficient details of methods and analysis provided to allow replication by others?

Yes

Reviewer Expertise:

My areas of research are about the War in Ukraine and its economic impacts, I lead a global coalition that has tracked COVID-19 recovery and is working on Russia-Ukraine war economic impacts. In terms of my academic training, I am a sociologist holding a PhD in sociology, with my research ranging from economic sociology to economic anthropology.

I confirm that I have read this submission and believe that I have an appropriate level of expertise to confirm that it is of an acceptable scientific standard.

# Response

**Published:** 2023-10-14

**Authors:** Silvia Mariela Méndez Prado

**Affiliations:** ESPOL university, Ecuador

We incorporated some of the main causal effects of the Russia-Ukraine war on the Ecuadorian economy, including the upsurge in fertilizer prices that can hinder one of the main economic sectors of Ecuador, banana exports. We also have added a reference explaining how income differences between males and females may influence their different perceptions regarding poverty and inflation because of the war.

# Reviewer report: Reviewer response for version 1

**DOI:** 10.5256/f1000research.144886.r204349

**Published:** 2023-09-18

**Authors:** Luisa Langer

**Affiliations:** 1 Universität Mannheim Fakultät für Betriebs­wirtschafts­lehre, Mannheim, Germany

This is a very good article, with a clear research aim, well-executed method, and interesting results.

 I have the following suggestions to the authors:

1. Title. It seems too broad. Authors could be more specific and refer to the University students' perceptions.
2. Introduction. Similar concern, in the fourth paragraph, the claims are too general. Authors refer to the citizens' economic perceptions and opinions, but the paper focuses on students' perceptions. This needs to be made clear especially when there are some differences between these groups (the sample of students is mostly non-working).
  Also, the last sentence in this paragraph requires mentioning some specific channels through which the conflict had an impact on the Ecuadorian economy.
3. Literature review. The discussion of various economic aspects in war times. I suggest the authors to consider adding some recent papers (if available) of the actual impact of the Russia-Ukraine war in other countries.
4. Methods. It would be interesting to know whether the same survey was used globally and in Ecuador, or if there was a need to adapt some questions. Moreover, can the authors provide some numbers regarding how many responses they received from ESPOL students and how many from other Universities? Similarly, are these Universities mostly public or private. 
5. Results. Some of the results might be a reflection of the country's economic characteristics. I suggest the authors to include some background and/or key statistics of Ecuador's main economic indicators. This could help understand the results better. For example, what were the inflation and poverty levels in the onset period of the war. Maybe those students' families were already impacted by those indicators and the war just aggravated those effects.

Overall, I enjoyed reading the paper and the insights provided helped to understand the war effects and perceptions.

Is the work clearly and accurately presented and does it cite the current literature?

Yes

If applicable, is the statistical analysis and its interpretation appropriate?

Yes

Are all the source data underlying the results available to ensure full reproducibility?

Yes

Is the study design appropriate and is the work technically sound?

Yes

Are the conclusions drawn adequately supported by the results?

Yes

Are sufficient details of methods and analysis provided to allow replication by others?

Yes

Reviewer Expertise:

Governance

I confirm that I have read this submission and believe that I have an appropriate level of expertise to confirm that it is of an acceptable scientific standard, however I have significant reservations, as outlined above.

# Response

**Published:** 2023-10-14

**Authors:** Silvia Mariela Méndez Prado

**Affiliations:** ESPOL university, Ecuador

1. Ok, adjustments have been made to the title.
2. We incorporated some references related to the perception of university students during past financial crises and rephrased some of the sentences to clarify the focus of the study, which is this cohort’s perceptions of the Russia-Ukraine war. We included some mechanisms channels in the previous paragraph to support the claim from the fourth paragraph.
3. Information on the impact of the Russia-Ukraine War can be found in the last paragraphs of each section from the literature review.
4. The survey applied in Ecuador was the same as from the one launched globally. We provide the total number of students that were registered during the term and those which participated according to the professors in charge of the courses. We also establish the number of responses outside ESPOL and the university categories they represent.
5. We reported the levels of unemployment, poverty threshold and inflation in the section of discussions to complement the analysis of results.

# Reviewer report: Reviewer response for version 1

**DOI:** 10.5256/f1000research.144886.r203783

**Published:** 2023-09-06

**Authors:** Ruth Endam Mbah

**Affiliations:** 1 Department of Business Administration, McPherson College, McPherson, Kansas, USA

This is a great article. However, I have the following suggestions:

1. I will propose a change of title for example; "Examining Economic Perceptions from LAC Region Towards an External Economic Event: Case Study of the Russia-Ukraine War" or "An Evaluation of Student's Perceptions of the Socio-economic Impact of the Russia-Ukraine War in Ecuador"
2. The abstract says "ANOVA confirms..." (the results section) but I don't see that methodology mentioned in the results or method section. I leave this to a statistician to affirm. 
3. Under Introduction, I would love to see citations for the claims made in paragraphs 2 & 3.
4. Under Hypotheses: I would love to see the null hypotheses stated since that is what we want to reject or fail to reject. I also think both hypotheses should be restated to make more sense. For instance when you say "differences in opinions would be found..."  the question that comes to my mind is differences in opinion about what? 
5. Under the methods section, it will be instrumental to know how many questionnaires were sent out initially. To know the response rate. Also, was the survey modeled after a previously published study, or is this one you created to allow for reliability and replication of the study? 
6. In the results section, I would have loved to the statistically insignificant figures for Supply Chain Disruption and Energy Prices as you did with the other variables. 
7. It would have been great to add COVID-19 as a variable given that most of the inflation, supply chain disruptions, etc already started during the pandemic 

I think this is a great work that needs some alterations. Their limitation section covers most of the concerns with might have but this sets a pace for future research.

Is the work clearly and accurately presented and does it cite the current literature?

Yes

If applicable, is the statistical analysis and its interpretation appropriate?

I cannot comment. A qualified statistician is required.

Are all the source data underlying the results available to ensure full reproducibility?

Yes

Is the study design appropriate and is the work technically sound?

Yes

Are the conclusions drawn adequately supported by the results?

Yes

Are sufficient details of methods and analysis provided to allow replication by others?

Yes

Reviewer Expertise:

International Trade, International Relations, Economic Development, Public Policy, Higher Education, Credit Management, Student Debt, Public Policy Theory

I confirm that I have read this submission and believe that I have an appropriate level of expertise to confirm that it is of an acceptable scientific standard, however I have significant reservations, as outlined above.

# Response

**Published:** 2023-10-14

**Authors:** Silvia Mariela Méndez Prado

**Affiliations:** ESPOL university, Ecuador

1. Ok.
2. It should be Kruskal-Wallis test, since that was the methodology employed.
3. We believe that the claims are supported through the hyperlinks that are included in the paragraphs.
4. We believe that the interpretation of the null hypotheses should be easily deduced by the reader, but a rewording of H1 and H2 was added.
5. We include the number of students that were enrolled in the 39 professors' courses, but we recognize the possibility for students to have enrolled in two courses, potentially duplicating entries, thus we cannot establish with certainty the total number of students. The survey was created by the COVID-19 social science lab, and we led the recruitment process in Ecuador.
6. Since results were not significant, we decided not to include their statistics as they are not informative, and we focused more on the variables that were significant.
7. This could be added as a limitation since the survey does not include an item related to this.
